# Disentangling drug contributions: anticholinergic burden in older adults linked to individual medications: a cross-sectional population-based study

**DOI:** 10.1186/s12877-023-04640-4

**Published:** 2024-01-10

**Authors:** Gauri Bhatkhande, Niteesh K. Choudhry, Mufaddal Mahesri, Nancy Haff, Julie C. Lauffenburger

**Affiliations:** 1 Division of Pharmacoepidemiology and Pharmacoeconomics, Department of Medicine, Brigham and Women’s Hospital and Harvard Medical School, 1620 Tremont Street, Suite 3030, Boston, MA 02120 USA; 2 Center for Healthcare Delivery Sciences (C4HDS), Department of Medicine, Brigham and Women’s Hospital and Harvard Medical School, Boston, MA USA

**Keywords:** Deprescribing, High-risk medications, Polypharmacy, Anticholinergic burden

## Abstract

**Background:**

Medications with potent anticholinergic properties have well-documented adverse effects. A high cumulative anticholinergic burden may arise from the concurrent use of multiple medications with weaker anticholinergic effects. We sought to identify patterns of high anticholinergic burden and associated patient characteristics.

**Methods:**

We identified patients aged ≥ 65 who filled ≥ 1 medication with anticholinergic adverse effects in 2019 and had a cumulative Anticholinergic Burden score (ACB) ≥ 4 (i.e., high anticholinergic burden) in a large US health insurer. We classified patients based on how they attained high burden, as follows: 1) only filling strong or moderate anticholinergic medications (i.e., ACB = 2 or 3, “moderate/strong”), 2) only filling lightly anticholinergic medications (i.e., ACB = 1, “light/possible”), and 3) filling any combination (“mix”). We used multinomial logistic regression to assess the association between measured patient characteristics and membership in the three anticholinergic burden classifications, using the moderate/strong group as the referent.

**Results:**

In total, 83,286 eligible patients with high anticholinergic burden were identified (mean age: 74.3 years (SD:7.1), 72.9% female). Of these, 4.5% filled only strong/moderate anticholinergics, 4.3% filled only light/possible anticholinergics, and the rest filled a mix (91.2%). Within patients in the mixed group, 64.3% of medication fills were for light/possible anticholinergics, while 35.7% were for moderate/strong anticholinergics. Compared with patients in the moderate/strong anticholinergics group, patients filling only light/possible anticholinergics were more likely to be older (adjusted Odds Ratio [aOR] per 1-unit of age: 1.06, 95%CI: 1.05–1.07), less likely to be female (aOR: 0.56, 95%CI: 0.50–0.62 vs. male), more likely to have comorbidities (e.g., heart failure aOR: 3.18, 95%CI: 2.70–3.74 or depression aOR: 1.20, 95%CI: 1.09–1.33 vs. no comorbidity), and visited fewer physicians (aOR per 1-unit of change: 0.98, 95%CI: 0.97–0.98). Patients in the mixed group were older (aOR per 1-unit of age: 1.02, 95%CI: 1.02–1.03) and less likely to be female (aOR: 0.89, 95%CI: 0.82–0.97 vs. male) compared with those filling moderate/strong anticholinergics.

**Conclusion:**

Most older adults accumulated high anticholinergic burden through a combination of light/possible and moderate/strong anticholinergics rather than moderate/strong anticholinergics, with light/possible anticholinergics being the major drivers of overall anticholinergic burden. These insights may inform interventions to improve prescribing in older adults.

**Supplementary Information:**

The online version contains supplementary material available at 10.1186/s12877-023-04640-4.

## Introduction

Many guidelines recommend limiting the use of medications with anticholinergic adverse effects in older adults due to their increased risk of cognitive impairment [1], dementia [2], confusion, and decline in physical function, including falls [3]. These risks are even more pronounced in older adults due to age-related changes, such as increased permeability of the blood brain barrier [4]. Older adults are also more likely to be exposed to medications with anticholinergic effects owing to multimorbidity and polypharmacy [5, 6].

While anticholinergics prescribed specifically for their anticholinergic effects are well-recognized as carrying these potential risks, high cumulative anticholinergic burden may also result from the concurrent use of multiple medications that individually have weaker anticholinergic effects [7, 8]. A high cumulative anticholinergic burden occurring from the use of multiple medications also confers an increased risk for adverse effects [9]. While there are several ways to characterize anticholinergic burden, common to most scales is classifying medications by their level of anticholinergic activity and summing these levels per patient, with a threshold for defining high burden [10–12]. For example, the Anticholinergic Burden score (ACB), derived from the Anticholinergic Cognitive Burden Score, is used to measure anticholinergic burden and ranges from 0 to 3 [12, 13]. Medications with ACB = 0 have no anticholinergic activity. Medications with ACB = 1 have light/possible anticholinergic effects, while those with ACB scores of 2 and 3 have established and clinically relevant anticholinergic effects.

A vast majority of prior work in this space has centered on assessing the impact of varying levels of anticholinergic burden on outcomes such as cognitive function, dementia, or fall risk [4, 14, 15]. Only a few studies have investigated the contribution of medications to anticholinergic burden and associated patient characteristics, especially within a contemporary context. It is widely recognized that strong anticholinergics are significant contributors to a high anticholinergic burden [16]. Emerging research has shed light on the pivotal role of light or weaker anticholinergics in the overall burden [8, 17]. A small study conducted in Australia, utilizing the Anticholinergic Drug Scale (ADS), found light anticholinergics to be major contributors to the overall burden [8]. Another small study conducted in Slovenia descriptively evaluated the characteristics for patients with a high overall burden [18]. While a few other studies have attempted to understand the proportion of prescribed medications with different levels of anticholinergic effect, fewer have delved into the intricacies of medication patterns among older adults that mirror real world scenarios and associated patient characteristics. A study in the US of nursing home residents evaluated patient characteristics associated with three different levels of cumulative anticholinergic burden in Medicare Part D beneficiaries [19]. However, there are very limited studies in the US of community-dwelling older adults who have examined the relative contribution of individual medications to this type of high anticholinergic burden, or patient characteristics associated with different types of burden. Accordingly, we sought to characterize the patterns of how community-dwelling older adults achieve high anticholinergic burden and patient characteristics associated with these classifications of burden, with the goal of informing future interventions.

## Methods

### Data source and patient population

We conducted a cross-sectional population-based study using de-identified claims for commercially insured beneficiaries from Optum’s de-identified Clinformatics® Data Mart Database that covers patients in all 50 states from 2018–2019 (in part to avoid any secular issues from the COVID-19 pandemic). These data include complete paid patient-level claims for inpatient and outpatient procedures, hospitalizations, emergency room and office visits, and outpatient prescription drug dispensations linked with enrollment data and eligibility data. The Mass General Brigham Institutional Review Board approved this study.

We identified patients ≥ 65 years of age as of January 1, 2019, who had continuous enrollment during the prior calendar year (i.e., 1/1/2018–1/1/2019), and who had filled ≥ 1 medication with anticholinergic side effects in calendar year 2019 to obtain an underlying denominator of patients filling these medications. Patients were excluded if they had missing age or sex.

### Anticholinergic burden

We measured anticholinergic burden using the latest version of the widely-used digital Anticholinergic Burden score (ACB) calculator [12, 13] as of 2019 [20]. The calculator is drawn from the Anticholinergic Cognitive Burden Scale and the German anticholinergic burden scale, which both assess the severity of negative anticholinergic effects of prescribed or over-the-counter medications on cognition in older adults and are some of the most commonly used metrics, particularly in claims [21]. This 4-point scale provides a list of 88 medications likely to have a negative impact on cognition and rates each drug with scores ranging from 1 (light/possible) to 3 (strong/high) anticholinergic burden based on serum anticholinergic activity and expert opinion. The individual scores of medications taken by the patient are summed to give the cumulative ACB score. Prior studies have shown ACB scores of ≥ 4 to be associated with worsened cognition and clinical outcomes [1], which we used here to identify patients with high anticholinergic burden.

To measure anticholinergic burden, we created a drug supply diary for each of the 88 medications beginning on 7/1/2018, stringing together fills based on dates of medication fills and days supplied, shifting the supply if there were overlapping fills (up to 30 days of stockpiling). Using the supply diary, we calculated the daily ACB score as a sum of the daily drug supply diaries between 1/1/2019–12/31/2019. For example, if a patient filled amantadine (ACB score = 2) and disopyramide (ACB score = 1) on 1/1/2019 with a days’ supply = 30 for both medications, the patient was given a daily ACB score = 3 for 1/1/2019–1/30/2019. We then took an average of the daily ACB scores over the calendar year to define patients’ level of anticholinergic burden.

To disentangle individual medication contributions, we categorized patients with an average ACB score ≥ 4 into 3 mutually exclusive categories based on how they attained high anticholinergic burden: 1) “moderate/strong anticholinergics”: filling only highly anticholinergic medications (i.e., only those with ACB = 2 or 3) 2) “light/possible anticholinergics”: filling only medications with low anticholinergic activity (i.e., ACB = 1) and 3) “mixed anticholinergics”: filling any combination of anticholinergic medications.

As secondary analyses, we further defined two subcategories for patients in the mixed group: ‘Majority light/possible anticholinergics: filling majority light/possible anticholinergics (i.e., the number of medications with ACB = 1 exceeded the number of medications with ACB = 2 and 3) and ‘Majority moderate/strong anticholinergics’: filling majority moderate/strong anticholinergics (i.e., the number of medications with ACB = 2 and 3 exceeded the number of medications with ACB = 1).

### Baseline covariates

We assessed patient covariates during the prior calendar year based on clinical knowledge and prior literature. Age, gender, and state/region of residence were obtained on the index date from claims data. We also measured the Gagne combined comorbidity score [22], the number of individual physicians associated with claims for the patient, number of emergency room (ER) visits, number of unique medications, number of hospitalizations, number of physician office visits, number of unique medication fills, and number of geriatrician office visits during the prior year using claims data; each of these variables were measured as continuous variables considering a one-unit change. Clinical comorbidities were assessed as dichotomous variables, where a value of 1 indicates presence of the comorbidity, and 0 indicates absence of comorbidity.

### Statistical analysis

We calculated the prevalence of high anticholinergic burden by the 3 categories of how they attained this burden among patients who filled anticholinergics. We computed descriptive statistics and compared baseline characteristics across the three groups using Chi-squared tests and ANOVA for categorical and continuous variables, respectively. We calculated the variance inflation factor (VIF), a measure of the amount of multicollinearity in regression analysis, to assess for multicollinearity between the independent variables. For each of the three groups, we calculated and plotted counts of the top 20 most prevalent medications with anticholinergic side effects. We used multinomial logistic regression to assess the association between the three classifications of high anticholinergic burden and patient characteristics, adjusting for all measured covariates. In specific, we compared light/possible anticholinergics and mixed anticholinergics versus moderate/strong anticholinergics as the referent group. All variables included in the models were significantly different across the three groups at baseline and had a VIF of less than 3, indicating the absence of multicollinearity. We used the Likelihood ratio test, Wald Chi-squared test, and McFadden pseudo-R-squared fit statistic to describe the model fit by comparing the full and nested models.

We also performed additional secondary and sensitivity analyses to explore certain patient subgroups in detail and included them in the Appendix. First, we analyzed the mixed group separately and compared patients within the mixed group to moderate/strong and light/possible groups respectively to better understand the group accounting for the vast majority of patients in our study by further defining the subcategories (majority light/possible anticholinergics, majority moderate/strong anticholinergics). For these analyses comparing two groups (e.g., comparisons within the mixed group), we used multivariable logistic regression. Second, we restricted the cohort further to patients with > 5 prescription fills to explore associations in patients with polypharmacy. Finally, we included the Gagne comorbidity index [22] instead of individual comorbidities in the models. All analyses used SAS Version 9.4 (SAS Institute Inc., Cary, NC).

## Results

**Fig. 1:**
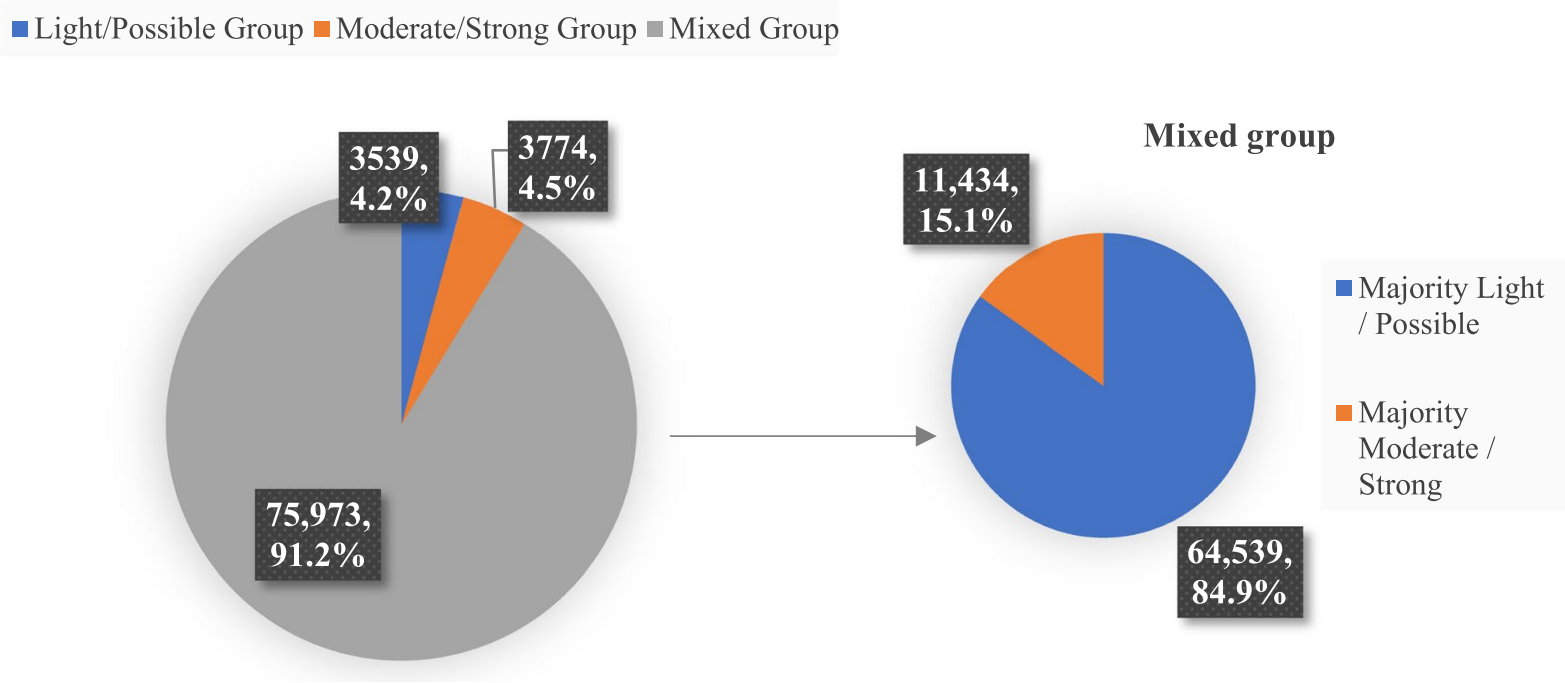
Patient-level patterns of achieving a high anticholinergic burden

Of 2,147,481 patients that filled ≥ 1 anticholinergic in 2019, 83,286 (3.9%) patients had a high anticholinergic burden (i.e., cumulative ACB score ≥ 4), with an average age of 74.3 years (SD 7.1) and were predominantly female (72.9%). 3774 patients (4.5%) only filled moderate/strong anticholinergic medications with a mean ACB score of 4.9 (SD:0.9). 3539 (4.2%) patients only filled light/possible anticholinergics with a mean ACB score of 4.6 (SD:0.6), while the remaining 75,973 (91.2%) patients in the mixed group filled a combination of moderate/strong and light/possible anticholinergics and had a mean ACB score of 5.2 (SD:1.2). Their baseline covariates are shown in Table 1. The distribution of patients across distinct categories and within the mixed group is shown in Fig. 1. Of patients in the mixed group, 64,539 patients (84.9%) filled mostly light/possible anticholinergics and 11,434 patients (15.1%) filled mostly moderate/strong anticholinergics. The baseline covariates of those in the stratified mixed group are shown in Appendix Table A in Additional file 1.

**Table 1:** Baseline demographic and clinical characteristics of anticholinergic users

**Characteristic**	0 < ACB < 4 Any anticholinergic (*N* = 2,064,195)	ACB ≥ 4				*P*-value
		All ACB ≥ 4 (*N* = 83,286)	Moderate/Strong: ACB = 2 and ACB = 3 drugs only (*N* = 3774)	Light/Possible: ACB = 1 drugs only (*N* = 3539)	Mixed: Any combination of ACB 1,2,3 (*N* = 75,973)	
Age, years (Mean, SD)	74.9(6.9)	74.3(7.1)	73.9(6.6)	78.5(7.3)	75.3(7.0)	< 0.001
Female (N, %)	1,240,075(60.1)	60,754(72.9)	2893(76.7)	2138(60.4)	55,723(73.4)	< 0.001
Region (N, %)						
Midwest	368,714(17.9)	16,568(19.9)	724(19.2)	786(22.2)	15,058(19.8)	< 0.001
Northeast	249,342(12.1)	9135(11.0)	465(12.3)	519(14.7)	8151(10.7)	
South	947,184(45.9)	41,079(49.3)	1651(43.7)	1644(46.5)	37,784(49.8)	
Other / Unknown	1993(0.1)	85(0.1)	< 10	< 10	77 (0.10)	
West	496,962(24.1)	16,419(19.7)	928(24.6)	588(16.6)	14,903(19.6)	
Healthcare utilization (in prior year), [Mean (SD)]						
No. of physicians	11.9(9.4)	16.6(11.7)	12.5(9.2)	17.6(12.1)	16.7(11.7)	< 0.001
No. of ER Visits	0.6(1.2)	1(1.9)	0.6(1.3)	1.1(1.8)	1.1(1.9)	< 0.001
No. of medications	10.3(5.9)	17.1(7.3)	12.3(5.9)	17.8(6.6)	17.3(7.3)	< 0.001
No. of geriatrician visits	0 (0.3)	0 (0.3)	0 (0.2)	0 (0.3)	0 (0.3)	0.1423
No. of hospitalizations	1.4(6.4)	3(11.4)	1.5(8.6)	3.9(13.1)	3.1(11.5)	< 0.001
No. of physician office visits	8.6(6.8)	11.8(8.6)	9.1(6.9)	10.8(8.6)	11.9(8.7)	< 0.001
No. of Rx fills	36.2(30.1)	82.5(57.9)	53.5(36.2)	104.7(77.4)	82.9(57.2)	< 0.001
Comorbidities (in prior year) (N, %)						
Atrial Fibrillation	320,716(15.5)	17,519(21.0)	175(4.6)	1530(43.2)	15,814(20.8)	< 0.001
Alcohol or drug dependence	220,575(10.7)	15,796(19.0)	527(14.0)	551(15.6)	14,718(19.4)	< 0.001
Alzheimer's disease/dementia	58,866(2.9)	4873(5.9)	150(4.0)	368(10.4)	4355(5.7)	< 0.001
Ischemic heart diseases	514,986(24.9)	27,189(32.6)	509(13.5)	1813(51.2)	24,867(32.7)	< 0.001
COPD	335,613(16.3)	23,812(28.6)	637(16.9)	1155(32.6)	22,020(29.0)	< 0.001
Dementia	268,054(13.0)	23,736(28.5)	695(18.4)	1449(40.9)	21,592(28.4)	< 0.001
Depression	430,019(20.8)	42,095(50.5)	1579(41.8)	1861(52.6)	38,655(50.9)	< 0.001
Diabetes	701,788(34.0)	35,937(43.1)	1218(32.3)	1721(48.6)	32,998(43.4)	< 0.001
Heart failure	273,983(13.3)	20,291(24.4)	239(6.3)	1661(46.9)	18,391(24.2)	< 0.001
Hyperlipidemia	1,480,958(71.7)	62,565(75.1)	2590(68.6)	2732(77.2)	57,243(75.4)	< 0.001
Hypertension	1,638,527(79.4)	72,128(86.6)	2633(69.8)	3248(91.8)	66,247(87.2)	< 0.001
MI	41,662(2.0)	2360(2.8)	26(0.7)	186(5.3)	2148(2.8)	< 0.001
Obesity	487,789(23.6)	26,129(31.4)	883(23.4)	994(28.1)	24,252(31.9)	< 0.001
Osteoporosis	232,235(11.3)	12,164(14.6)	525(13.9)	466(13.2)	11,173(14.7)	0.019
PVD	266,639(12.9)	15,344(18.4)	417(11.0)	941(26.6)	13,986(18.4)	< 0.001
Rheumatic heart disease	84,831(4.1)	4628(5.6)	84(2.2)	339(9.6)	4205(5.5)	< 0.001
Renal dysfunction	26,624(1.3)	1519(1.8)	36(1.0)	104(2.9)	1379(1.8)	< 0.001
Sleep Apnea	247,138(12.0)	16,596(19.9)	479(12.7)	809(22.9)	15,308(20.2)	< 0.001
Smoking history	461,101(22.3)	25,873(31.1)	864(22.9)	1106(31.3)	23,903(31.5)	< 0.001
Stable Angina	122,328(5.9)	7123(8.6)	103(2.7)	499(14.1)	6521(8.6)	< 0.001
Stroke or TIA	103,352(5.0)	6470(7.8)	176(4.7)	328(9.3)	5966(7.9)	< 0.001
Unstable Angina	34,590(1.7)	2101(2.5)	22(0.6)	147(4.2)	1932(2.5)	< 0.001
Cardiac valve disorder	85,317(4.1)	4649(5.6)	69(1.8)	368(10.4)	4212(5.5)	< 0.001

*Abbreviations*: *ACB* Anticholinergic burden score (2019), *SD* Standard deviation, *ER* Emergency Room, *COPD* Chronic obstructive pulmonary disease, *MI* Myocardial infarction, *PVD* Peripheral vascular disease, *TIA* Transient ischaemic attack

The distributions of counts of individual anticholinergic medication fills are shown by group in the Appendix Figure A. Overall, 2,506,084 anticholinergics were prescribed with a cumulative ACB score of 432,419. Of these, 1,634,899 (65.2%) were light/possible anticholinergics with an ACB score of 1, 259,405 (10.4%) had an ACB score of 2 and 611,780 (24.4%) had an ACB score of 3. Of the 2,506,084 anticholinergics, 164,751 (6.6%) anticholinergics were prescribed in the light group, 55,025 (2.2%) anticholinergics were prescribed in the moderate/strong group, and 2,286,308 (91.2%) were prescribed in the mixed group. Of the 2,286,308 anticholinergics filled in the mixed group, 1,470,148 (64.3%) were light/possible anticholinergics with an ACB score of 1, 244,521 (10.7%) were moderate anticholinergics with an ACB score of 2, and 571,639 (25.0%) were strong anticholinergics with an ACB score of 3. Therefore, majority of the anticholinergics contributing to the mixed group were in fact light anticholinergics with an ACB score of 1.

**Fig. 2:**
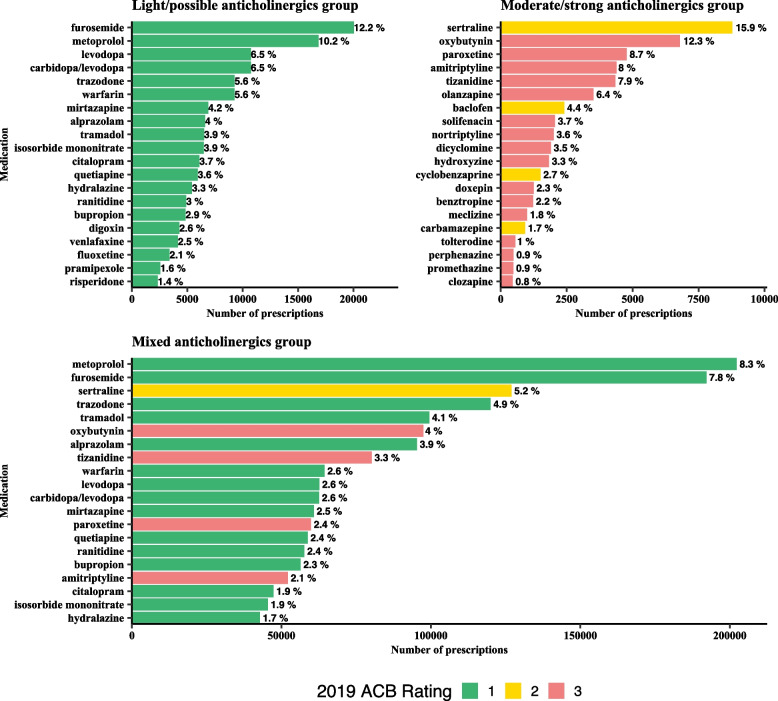
20 most common medications with anticholinergic properties among those with high anticholinergic burden Abbreviations: ACB, Anticholinergic Burden score (2019). For each group, the overall number of anticholinergic prescriptions are shown, with percentages indicating the percentage of patients in that group filling that medication. Moderate/strong anticholinergics: ACB = 2 and ACB = 3 drugs only; Light/possible anticholinergics: ACB = 1 drugs only; Mixed anticholinergics: Any combination of ACB = 1, 2, or 3

The most common medications within each group are shown in Fig. 2. Overall, cardiovascular medications (*N* = 62,292, 37.8%) were the most prescribed among patients in the light/possible group, followed by psychotropic medications (*N* = 49,478, 30.0%). Among patients in the moderate/strong group, the most common medications were psychotropic medications (*N* = 27,041, 49.1%). Finally, the most common medications in the mixed group were light/possible anticholinergics, with the most prescribed medications being psychotropics (*N* = 6,77,688, 29.6%) and cardiovascular medications (*N* = 5,47,192, 23.9%).

**Fig. 3:**
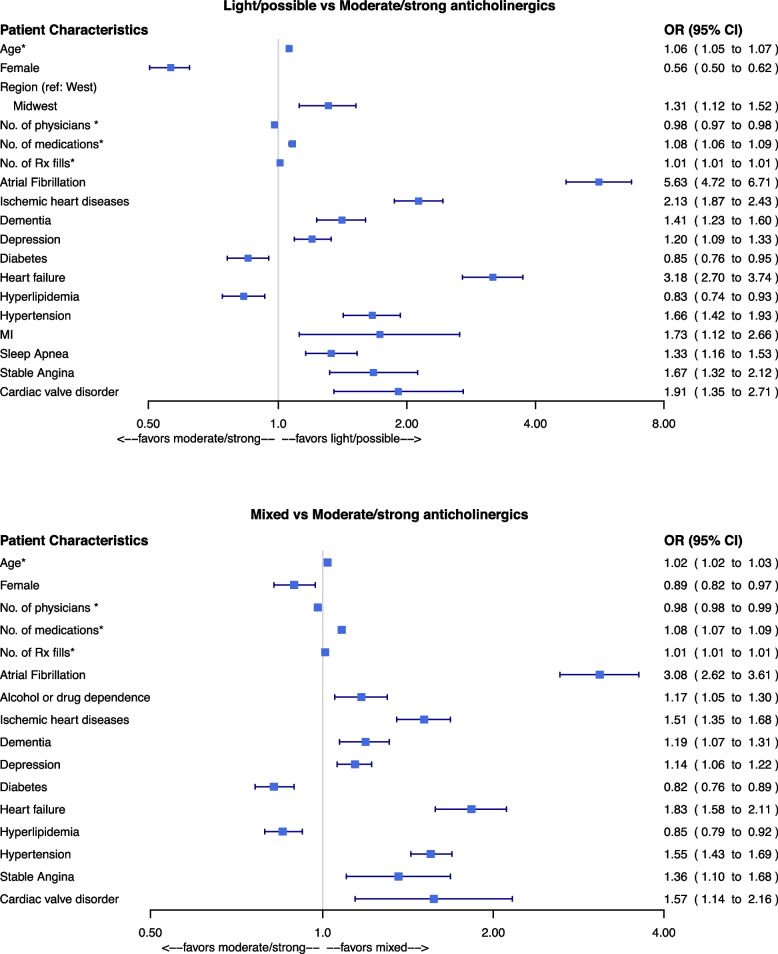
Patient characteristics associated with different categories of high anticholinergic burden Note: Moderate/strong anticholinergics: ACB = 2 and ACB = 3 drugs only; Light/possible anticholinergics: ACB = 1 drugs only; Mixed anticholinergics: Any combination of ACB = 1, 2, or 3. * indicates per 1-unit change. # This figure contains significant associations. The overall model is included in the Appendix. Model fit statistics: Wald Chi-squared statistic = 4903.4491, *P*-value: < 0.001; Likelihood ratio test Chi-squared statistic = 5860.0867, *P*-value: < 0.001; R-squared statistic = 0.1328. Abbreviations: ER, Emergency Room; Rx, Medical prescription; COPD, Chronic obstructive pulmonary disease; MI, Myocardial infarction

The multinomial regression model comparing light/possible anticholinergics group and mixed group with the referent moderate/strong anticholinergics group is shown in Fig. 3. For illustrative purposes, we have included the significant variables in Fig. 3 and the overall model results in Appendix Figure B. Using a threshold of less than 3.0, we found no significant collinearity between the variables in the regression model. Compared with the moderate/strong anticholinergics group in adjusted models, patients who filled light/possible anticholinergics were more likely to be older (adjusted Odds Ratio [aOR]: 1.06, 95%CI: 1.05–1.07), less likely to be female (aOR: 0.56, 95%CI: 0.50–0.62; referent group = male), had fewer physicians (aOR per 1-unit change: 0.98, 95%CI: 0.97–0.98), filled more medications (aOR per 1-unit change: 1.08, 95%CI: 1.06–1.09), and often had more comorbidities (Fig. 3). Compared with the moderate/strong anticholinergics group, patients in the light/possible group had more comorbidities with mainly cardiovascular conditions (e.g., heart failure aOR: 3.18, 95%CI:2.70–3.74, ischemic heart disease aOR: 2.13, 95%CI: 1.87–2.43, depression aOR: 1.20, 95%CI: 1.09–1.33, hypertension aOR: 1.66, 95%CI:1.42–1.93; referent = not having the comorbidity), and were less likely to be diagnosed with diabetes and hyperlipidemia (diabetes aOR: 0.85, 95%CI: 0.76–0.95, hyperlipidemia aOR: 0.83, 95%CI: 0.74–0.93; referent = not having the comorbidity).

Conversely, compared with the moderate/strong group, patients who filled mixed anticholinergics were more likely to be older (aOR per 1-unit change: 1.02, 95%CI: 1.02–1.03) and less likely to be female (aOR: 0.89, 95%CI: 0.82–0.97; referent group = male), as shown in adjusted models (Fig. 3). Compared with the moderate/strong group, patients in the mixed anticholinergic group also had fewer physicians (aOR per 1-unit change: 0.98, 95%CI: 0.98–0.99) and had a higher likelihood of having most comorbidities (e.g., heart failure aOR: 1.83, 95%CI: 1.58–2.11, ischemic heart disease aOR: 1.51, 95%CI: 1.35–1.68, depression aOR: 1.14, 95%CI: 1.06–1.22, hypertension aOR: 1.55, 95%CI: 1.43–1.69; referent = not having the comorbidity) except for diabetes (OR: 0.82, 95%CI: 0.76–0.89) and hyperlipidemia (aOR:0.85, 95%CI: 0.79–0.92).

Our sensitivity analyses supported the robustness of the main findings and largely remained unchanged. Compared with majority moderate/strong group, patients who filled majority light anticholinergics in the mixed group showed similar associations as the overarching light/possible vs moderate/strong comparisons (Appendix Tables B, C, and D). Estimates also did not change appreciably when adjusting for Gagne comorbidity index rather than individual comorbidities (Appendix Table E) or when restricting the cohort to patients with polypharmacy (Appendix Table F).

## Discussion

In a nationwide cohort of older adults with high anticholinergic burden, we found that the vast majority of individuals achieved high anticholinergic burden through filling a combination of light/possible and moderate/strong anticholinergics, driven by light/possible anticholinergic prescriptions, rather than medications with moderate/strong anticholinergic adverse effects. Even a majority of patients in the mixed group actually filled mostly light/possible anticholinergics. Individuals filling only moderate/strong anticholinergics were more likely to be younger, female, and had fewer comorbidities than the other two groups.

To our knowledge, no prior study has sought to identify specific medications and patterns behind how community-dwelling US older adults accumulate high anticholinergic burden. Prior studies have focused on patients with specific conditions, [23, 24] only descriptively assessed patient attributes and burden, [18] had a relatively small sample size, [18, 25, 26] examined populations outside the US, [17, 18, 25], examined nursing home populations [19], or analyzed the overall total burden score without disentangling the underlying drug composition [16, 27, 28]. For instance, a study in the US conducted by Niznik et al. found that a high anticholinergic burden was the result of one strong anticholinergic medication rather than a cumulative effect but was performed in the nursing home setting [19]. By contrast, our study found that a large portion of the anticholinergic burden is a result of cumulative effect of light/possible and moderate/strong anticholinergics rather than solely strong anticholinergics. Some of our findings are consistent with another study in Australia which found that light anticholinergics contributed substantially (64–70%) to the total anticholinergic load in patients [8]. However, our findings are not directly comparable as they did not examine individual filling patterns and most patients also had probable dementia.

Our finding that patients filling moderate/strong anticholinergics were more likely to be women corroborates previous reports that have uncovered similar associations between sex and anticholinergic prescriptions [29, 30]. This finding may be explained by the increased likelihood for women to receive prescriptions for conditions like bladder incontinence or depression that are more common among women [17, 31, 32] and are known drivers of increased anticholinergic medication use [29]. Moreover, some studies suggest that women may be more likely to experience polypharmacy compared to men. In another study with older adults newly prescribed cholinesterase inhibitor therapy, female sex and having multiple physicians were also contributors to high anticholinergic burden [30].

One thing of note in our study is that most patients in the mixed group filling combinations of anticholinergics also filled largely light/possible anticholinergics, and they also filled more light/possible anticholinergics rather than moderate/strong anticholinergics. On balance, patients filling only light/possible anticholinergics tended to be older than other groups. There are several potential explanations for these findings. First, physicians may be exercising caution when prescribing strong anticholinergics or intentional in choosing medications with low anticholinergic activity for this age group. Second, patients in the light/possible group were more likely to have cardiovascular conditions such as atrial fibrillation or heart failure and accordingly had more prescriptions for these conditions; many of these commonly prescribed cardiovascular medications have light or possible anticholinergic activity. Accordingly, a number of older adults may experience cumulative anticholinergic burden related to cardiovascular medications. Other studies have also found cardiovascular medications to be prominent contributors to the anticholinergic burden, specifically light/possible anticholinergics, [8, 17] and are often prescribed simultaneously with other anticholinergic medications such as bladder antimuscarinics and antidepressants [27]. In a German study of 3189 patients, cardiovascular medications contributed highly to the light anticholinergics [17]. While the burden of these medications may be light and these cardiovascular medications may be difficult to clinically modify, clinicians may then need to exercise caution while prescribing other non-cardiovascular medications to older patients with common cardiovascular conditions.

Our study has several implications for clinical care and deprescribing intervention design. One key observation is that the majority of patients attain a substantial anticholinergic burden using a combination of light/possible and moderate/strong medications, rather than solely moderate/strong anticholinergic medications, which is previously what many have focused on. This observation is important because while having overall high anticholinergic burden has been associated with adverse cognitive outcomes, the level of risk itself may also depend on the individual medications that contribute to the overall cumulative score [33]. For instance, Green et al. reported that strongly anticholinergic medications were associated with the highest increase in risk of falls or fall-related injuries, followed by multiple light anticholinergics [34]. While current deprescribing interventions and tools mainly focus on strong anticholinergics, it may be important to consider the cumulative effect of multiple light/possible anticholinergics [35]. This finding underscores that deprescribing efforts may need to focus on more light/possible anticholinergics rather than just strong anticholinergics, particularly given the overall volume of individuals achieving high anticholinergic burden through cumulative prescribing of light/possible anticholinergics. One example solution may also be through the provision of enhanced electronic health record tools for providers that provide more nuance about specific anticholinergic medications that may be easier targets for medication optimization.

This study has some limitations. First, we relied on insurance claims data to define prescription filling and cannot identify over-the-counter medications as well as medications that patients were prescribed but did not fill. We also did not evaluate medication filling patterns with a comparator having ACB scores < 4, as those patients may be fundamentally different and less comparable. The score may have undergone some changes in the past few years in terms of medication classifications. Our study also focused on patients with commercial insurance and may not be fully generalizable to other older adults, but we expect these individuals to be even healthier on average than other older adults.

## Conclusion

Most older adults experiencing high anticholinergic burden are filling a combination of moderate/strong and light/possible anticholinergics, with light/possible anticholinergics being the major contributors of this group. Individuals who exclusively filled moderate/strong anticholinergics were more likely to be women, had more physicians and hospitalizations, while those on light/possible anticholinergics tended to be older and had the highest number of medications for cardiovascular conditions. By recognizing these distinct groups, we can better tailor interventions to meet their specific needs and maximize our efforts towards improving prescribing practices.

### Supplementary Information

**Additional file 1: Appendix Table A.** Baseline demographic and clinical characteristics of anticholinergic users: Stratification of the mixed group. **Appendix Figure A**. Distribution of individual anticholinergic prescription medication fills across groups. **Appendix Figure B. **Patient characteristics associated with different categories of high anticholinergic burden: Full set of covariates. **Appendix Table B. **Patient characteristics associated with different categories of patients in the mixed group. **Appendix Table C. **Patient characteristics associated with different categories of patients in the mixed group compared with the moderate/strong group. **Appendix Table D. **Patient characteristics associated with different categories of patients in the mixed group compared with light/possible group. **Appendix Table E. **Patient characteristics associated with different categories of high anticholinergic burden adjusted for Gagne comorbidity index rather than individual comorbidities. **Appendix Table F.** Patient characteristics associated with different categories of high anticholinergic burden in patients with >5 prescription fills.

## Data Availability

The datasets generated and/or analysed during the current study are not publicly available due to the nature of the human subjects’ data obtained from Optum’s de-identified Clinformatics® Data Mart Database but are available from the corresponding author on reasonable request.

## Abbreviations

ACB: Anticholinergic burden score
ER: Emergency room
Rx: Medical prescription
COPD: Chronic obstructive pulmonary disease
MI: Myocardial infarction
PVD: Peripheral vascular disease
TIA: Transient ischemic attack
