# Supplementary material for: Disentangling drug contributions: anticholinergic burden in older adults linked to individual medications: a cross-sectional population-based study
Source: BMC Geriatr. 2024 Jan 10;24:44. doi: 10.1186/s12877-023-04640-4 (PMC10782746; doi:10.1186/s12877-023-04640-4)
Supplement: Supplementary file 1 — Additional file 1: Appendix Table A. Baseline demographic and clinical characteristics of anticholinergic users: Stratification of the mixed group. Appendix Figure A. Distribution of individual anticholinergic prescription medication fills across groups. Appendix Figure B. Patient characteristics associated with different categories of high anticholinergic burden: Full set of covariates. Appendix Table B. Patient characteristics associated with different categories of patients in the mixed group. Appendix Table C. Patient characteristics associated with different categories of patients in the mixed group compared with the moderate/strong group. Appendix Table D. Patient characteristics associated with different categories of patients in the mixed group compared with light/possible group. Appendix Table E. Patient characteristics associated with different categories of high anticholinergic burden adjusted for Gagne comorbidity index rather than individual comorbidities. Appendix Table F. Patient characteristics associated with different categories of high anticholinergic burden in patients with >5 prescription fills. [file 12877_2023_4640_MOESM1_ESM.docx]

**APPENDIX FILE 1**

**Appendix Table A: Baseline demographic and clinical characteristics of anticholinergic users: Stratification of the mixed group**

| **Characteristic** | **Strong/Moderate anticholinergics**  **(N = 3,774)** | **Light/Possible anticholinergics**  **(N = 3,539)** | **Mixed (N = 75,973)** | | **P-value** |
| --- | --- | --- | --- | --- | --- |
|  |  |  | **Majority Light anticholinergics (N = 64,539)** | **Majority Strong anticholinergics (N = 11,434)** |  |
| Age, years (Mean, SD) | 73.9(6.6) | 78.5(7.3) | 75.6(7.1) | 74(6.7) | <0.001 |
| Female (N, %) | 2893(76.7) | 2138(60.4) | 46830(72.6) | 8893(77.8) | <0.001 |
| **Region (N, %)** | | | | | <0.001 |
| Midwest | 724(19.2) | 786(22.2) | 12884(20) | 2174(19.1) |  |
| Northeast | 465(12.3) | 519(14.7) | 7036(11) | 1115(9.8) |  |
| South | 1651(43.7) | 1644(46.5) | 32101(49.8) | 5683(49.8) |  |
| Other / Unknown | <10 | <10 | 67 (0.1) | 10 (0.1) |  |
| West | 928(24.6) | 588(16.6) | 12451(19.3) | 2452(21.5) |  |
| **Healthcare utilization (in prior year), [Mean (SD)]** | | | | | |
| No. of physicians | 12.5(9.2) | 17.6(12.1) | 16.8(11.8) | 16.6(11.6) | <0.001 |
| No. of ER Visits | 0.6(1.3) | 1.1(1.8) | 1.1(1.9) | 1.0(2.1) | <0.001 |
| No. of medications | 12.3(5.9) | 17.8(6.6) | 17.4(7.4) | 17.0(7.4) | <0.001 |
| No. of geriatrician visits | 0 (0.2) | 0 (0.3) | 0 (0.3) | 0 (0.3) | 0.192 |
| No. of hospitalizations | 1.5(8.6) | 3.9(13.1) | 3.2(12) | 2.7(8.8) | <0.001 |
| No. of physician office visits | 9.1(6.9) | 10.8(8.6) | 11.9(8.7) | 12.1(8.8) | <0.001 |
| No. of Rx fills | 53.5(36.2) | 104.7(77.4) | 84.2(58.7) | 76.3(48.4) | <0.001 |
| **Comorbidities (in prior year) (N, %)** | | | | | |
| Atrial Fibrillation | 175(4.6) | 1530(43.2) | 14424(22.4) | 1390(12.2) | <0.001 |
| Alcohol or drug dependence | 527(14.0) | 551(15.6) | 12285(19.1) | 2433(21.3) | <0.001 |
| Alzheimer's disease/dementia | 150(4.0) | 368(10.4) | 3763(5.9) | 592(5.2) | <0.001 |
| Ischemic heart diseases | 509(13.5) | 1813(51.2) | 22012(34.2) | 2855(25) | <0.001 |
| COPD | 637(16.9) | 1155(32.6) | 18983(29.5) | 3037(26.6) | <0.001 |
| Dementia | 695(18.4) | 1449(40.9) | 18653(28.9) | 2939(25.7) | <0.001 |
| Depression | 1579(41.8) | 1861(52.6) | 32515(50.4) | 6140(53.7) | <0.001 |
| Diabetes | 1218(32.3) | 1721(48.6) | 28459(44.1) | 4539(39.7) | <0.001 |
| Heart failure | 239(6.3) | 1661(46.9) | 16602(25.8) | 1789(15.7) | <0.001 |
| Hyperlipidemia | 2590(68.6) | 2732(77.2) | 48824(75.7) | 8419(73.7) | <0.001 |
| Hypertension | 2633(69.8) | 3248(91.8) | 56739(88) | 9508(83.2) | <0.001 |
| MI | 26(0.7) | 186(5.3) | 1909(3) | 239(2.1) | <0.001 |
| Obesity | 883(23.4) | 994(28.1) | 20729(32.2) | 3523(30.9) | <0.001 |
| Osteoporosis | 525(13.9) | 466(13.2) | 9355(14.5) | 1818(15.9) | <0.001 |
| PVD | 417(11.0) | 941(26.6) | 12162(18.9) | 1824(16) | <0.001 |
| Rheumatic heart disease | 84(2.2) | 339(9.6) | 3766(5.9) | 439(3.9) | <0.001 |
| Renal dysfunction | 36(1.0) | 104(2.9) | 1229(1.9) | 150(1.4) | <0.001 |
| Sleep Apnea | 479(12.7) | 809(22.9) | 13212(20.5) | 2096(18.4) | <0.001 |
| Smoking history | 864(22.9) | 1106(31.3) | 20291(31.5) | 3612(31.6) | <0.001 |
| Stable Angina | 103(2.7) | 499(14.1) | 5818(9.1) | 703(6.2) | <0.001 |
| Stroke or TIA | 176(4.7) | 328(9.3) | 5181(8.1) | 785(6.9) | <0.001 |
| Unstable Angina | 22(0.6) | 147(4.2) | 1732(2.7) | 200(1.8) | <0.001 |
| Cardiac valve disorder | 69(1.8) | 368(10.4) | 3806(5.9) | 406(3.6) | <0.001 |
| Abbreviations: ACB, Anticholinergic burden score (2019); SD, Standard deviation; ER, Emergency Room; COPD, Chronic obstructive pulmonary disease; MI, Myocardial infarction; PVD, Peripheral vascular disease; TIA, Transient ischaemic attack | | | | | |
|  | | | | | |

**Appendix Figure A**: **Distribution of individual anticholinergic prescription medication fills across groups**

­

**Appendix Figure B: Patient characteristics associated with different categories of high anticholinergic burden: Full set of covariates**

**
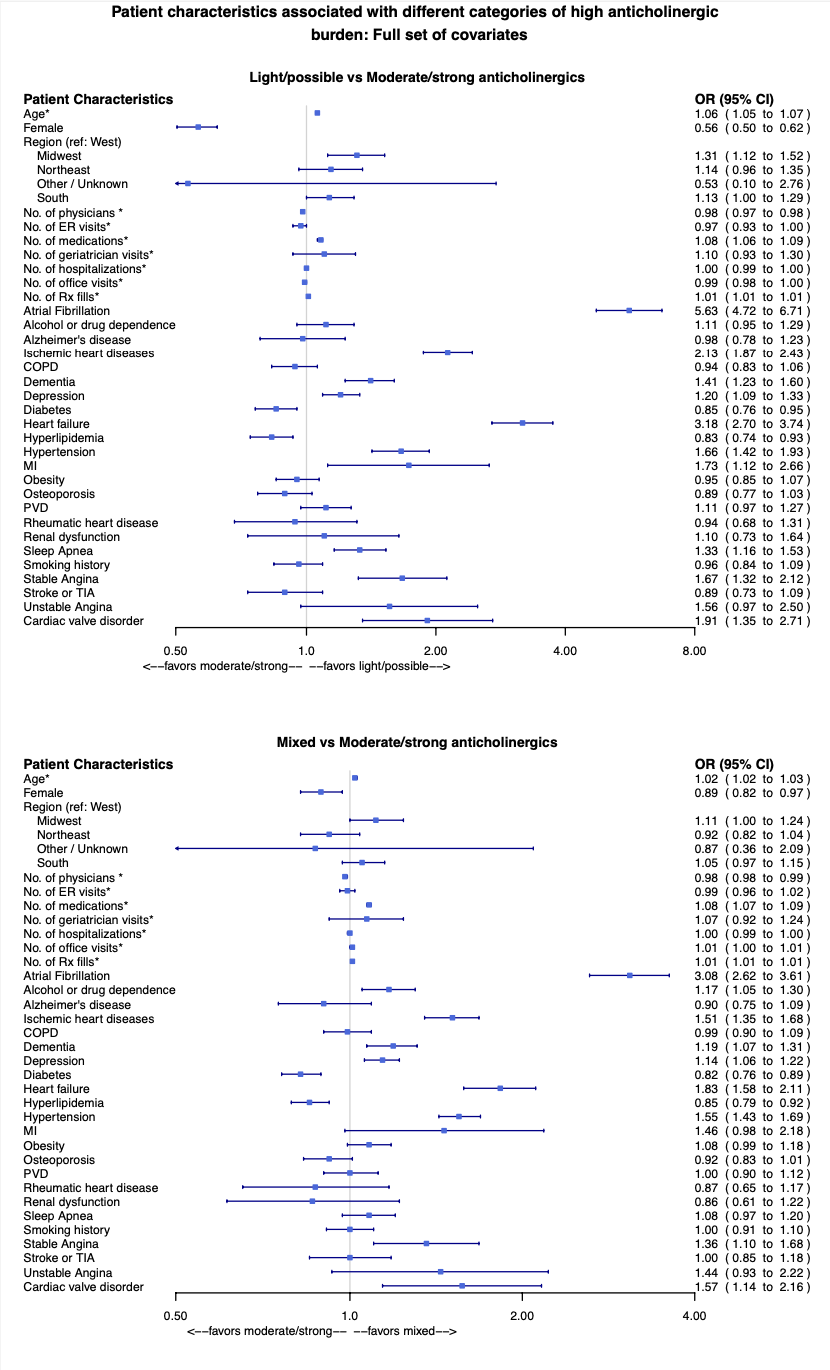
**

Note: Moderate/strong anticholinergics: ACB=2 and ACB=3 drugs only; Light/possible anticholinergics: ACB=1 drugs only; Mixed anticholinergics: Any combination of ACB=1, 2, or 3.

* indicates per 1-unit change.

Model fit statistics: Wald Chi-squared statistic = 4903.4491, P-value: <0.001; Likelihood ratio test Chi-squared statistic = 5860.0867, P-value: <0.001; R-squared statistic = 0.1328

Abbreviations: ER, Emergency Room; Rx, Medical prescription; COPD, Chronic obstructive pulmonary disease; MI, Myocardial infarction; PVD, Peripheral vascular disease; TIA, Transient ischemic attack

**Appendix Table B: Patient characteristics associated with different categories of patients in the mixed group.**

| **Characteristic** | **Majority light (n= 64,539) vs. Majority moderate/strong (n= 11434)** | | |
| --- | --- | --- | --- |
|  | **Odds Ratio** | **95% Confidence Limits** | |
| Age* | 1.02 | 1.02 | 1.03 |
| Female | 0.83 | 0.79 | 0.88 |
| **Region (ref: West)** | | | |
| Midwest | 1.09 | 1.02 | 1.16 |
| Northeast | 1.11 | 1.03 | 1.20 |
| Other / Unknown | 1.25 | 0.64 | 2.44 |
| South | 1.06 | 1.01 | 1.12 |
| **Healthcare utilization (in prior year)** | | | |
| No. of physicians* | 0.99 | 0.99 | 0.99 |
| No. of ER visits* | 0.99 | 0.98 | 1.00 |
| No. of unique medications* | 0.99 | 0.99 | 1.00 |
| No. of geriatrician visits* | 1.02 | 0.96 | 1.09 |
| No. of hospitalizations* | 1.00 | 1.00 | 1.01 |
| No. of physician office visits* | 1.59 | 1.49 | 1.69 |
| No. of Rx fills* | 0.96 | 0.90 | 1.01 |
| **Comorbidities (in prior year)** | | | |
| Atrial Fibrillation | 1.19 | 1.13 | 1.26 |
| Alcohol or drug dependence | 1.03 | 0.98 | 1.09 |
| Alzheimer's disease/dementia | 1.02 | 0.97 | 1.08 |
| Ischemic heart diseases | 0.89 | 0.85 | 0.93 |
| COPD | 1.05 | 1.00 | 1.10 |
| Dementia | 1.39 | 1.31 | 1.48 |
| Depression | 0.99 | 0.94 | 1.04 |
| Diabetes | 1.20 | 1.13 | 1.27 |
| Heart failure | 1.01 | 0.88 | 1.17 |
| Hyperlipidemia | 1.05 | 1.00 | 1.10 |
| Hypertension | 0.95 | 0.90 | 1.00 |
| MI | 0.99 | 0.94 | 1.05 |
| Obesity | 1.04 | 0.91 | 1.19 |
| Osteoporosis | 1.20 | 1.01 | 1.43 |
| PVD | 1.10 | 1.04 | 1.16 |
| Rheumatic heart disease | 0.98 | 0.93 | 1.03 |
| Renal dysfunction | 1.17 | 1.07 | 1.28 |
| Sleep Apnea | 1.01 | 0.93 | 1.09 |
| Smoking history | 1.13 | 0.97 | 1.33 |
| Stable Angina | 1.22 | 1.06 | 1.40 |
| Stroke or TIA | 1.59 | 1.49 | 1.69 |
| Unstable Angina | 0.96 | 0.90 | 1.01 |
| Cardiac valve disorder | 0.88 | 0.79 | 0.97 |

* indicates per 1-unit change.

Abbreviations: ER, Emergency Room; Rx, Medical prescription; COPD, Chronic obstructive pulmonary disease; MI, Myocardial infarction; PVD, Peripheral vascular disease; TIA, Transient ischemic attack

**Appendix Table C: Patient characteristics associated with different categories of patients in the mixed group compared with the moderate/strong group.**

| **Characteristic** | **Majority light (n= 64,539) vs. Moderate/strong (n= 3,774)** | | | **Majority Moderate/strong (n= 11,434) vs. Moderate/strong (n= 3,774)** | | |
| --- | --- | --- | --- | --- | --- | --- |
|  | **Odds Ratio** | **95% Confidence Limits** | | **Odds Ratio** | **95% Confidence Limits** | |
| Age* | 1.03 | 1.02 | 1.03 | 1.00 | 1.00 | 1.01 |
| Female | 0.87 | 0.80 | 0.94 | 1.04 | 0.95 | 1.14 |
| **Region (ref: West)** | | | | | | |
| Midwest | 1.14 | 1.02 | 1.26 | 1.04 | 0.93 | 1.17 |
| Northeast | 0.94 | 0.83 | 1.06 | 0.85 | 0.74 | 0.97 |
| Other / Unknown | 0.92 | 0.38 | 2.21 | 0.73 | 0.26 | 2.08 |
| South | 1.07 | 0.98 | 1.16 | 1.00 | 0.91 | 1.11 |
| **Healthcare utilization (in prior year)** | | | | | | |
| No. of physicians* | 0.98 | 0.98 | 0.99 | 0.99 | 0.99 | 1.00 |
| No. of ER visits* | 0.99 | 0.96 | 1.01 | 0.99 | 0.96 | 1.03 |
| No. of unique medications* | 1.08 | 1.07 | 1.09 | 1.09 | 1.08 | 1.10 |
| No. of geriatrician visits* | 1.07 | 0.92 | 1.24 | 1.05 | 0.90 | 1.22 |
| No. of hospitalizations* | 1.00 | 0.99 | 1.00 | 1.00 | 0.99 | 1.00 |
| No. of physician office visits* | 1.01 | 1.00 | 1.01 | 1.01 | 1.00 | 1.01 |
| No. of Rx fills* | 1.01 | 1.01 | 1.01 | 1.01 | 1.00 | 1.01 |
| **Comorbidities (in prior year)** | | | | | | |
| Atrial Fibrillation | 3.25 | 2.77 | 3.82 | 2.05 | 1.73 | 2.42 |
| Alcohol or drug dependence | 1.16 | 1.04 | 1.29 | 1.21 | 1.08 | 1.37 |
| Alzheimer's disease/dementia | 0.90 | 0.74 | 1.08 | 1.02 | 0.83 | 1.26 |
| Ischemic heart diseases | 1.55 | 1.39 | 1.72 | 1.30 | 1.15 | 1.46 |
| COPD | 1.00 | 0.91 | 1.10 | 0.97 | 0.87 | 1.08 |
| Dementia | 1.19 | 1.07 | 1.32 | 1.16 | 1.04 | 1.30 |
| Depression | 1.11 | 1.03 | 1.19 | 1.25 | 1.16 | 1.36 |
| Diabetes | 0.83 | 0.77 | 0.90 | 0.79 | 0.73 | 0.86 |
| Heart failure | 1.91 | 1.65 | 2.20 | 1.37 | 1.17 | 1.60 |
| Hyperlipidemia | 0.85 | 0.79 | 0.93 | 0.86 | 0.79 | 0.94 |
| Hypertension | 1.61 | 1.48 | 1.75 | 1.35 | 1.23 | 1.48 |
| MI | 1.47 | 0.98 | 2.20 | 1.45 | 0.95 | 2.21 |
| Obesity | 1.09 | 1.00 | 1.19 | 1.04 | 0.95 | 1.14 |
| Osteoporosis | 0.91 | 0.82 | 1.00 | 0.96 | 0.86 | 1.07 |
| PVD | 1.00 | 0.89 | 1.11 | 1.01 | 0.89 | 1.14 |
| Rheumatic heart disease | 0.88 | 0.66 | 1.18 | 0.85 | 0.62 | 1.16 |
| Renal dysfunction | 0.88 | 0.62 | 1.24 | 0.73 | 0.50 | 1.06 |
| Sleep Apnea | 1.10 | 0.99 | 1.22 | 1.01 | 0.90 | 1.13 |
| Smoking history | 1.00 | 0.91 | 1.10 | 1.02 | 0.92 | 1.13 |
| Stable Angina | 1.39 | 1.13 | 1.72 | 1.19 | 0.95 | 1.49 |
| Stroke or TIA | 1.00 | 0.85 | 1.18 | 1.00 | 0.84 | 1.19 |
| Unstable Angina | 1.46 | 0.95 | 2.27 | 1.29 | 0.82 | 2.04 |
| Cardiac valve disorder | 1.61 | 1.17 | 2.21 | 1.32 | 0.94 | 1.86 |

* indicates per 1-unit change.

Abbreviations: ER, Emergency Room; Rx, Medical prescription; COPD, Chronic obstructive pulmonary disease; MI, Myocardial infarction; PVD, Peripheral vascular disease; TIA, Transient ischemic attack

**Appendix Table D: Patient characteristics associated with different categories of patients in the mixed group compared with light/possible group.**

| **Characteristic** | **Majority Light (n= 64,539) vs. Light/Possible (n= 3,539)** | | | **Majority Moderate/strong (n= 11,434) vs. Light/Possible (n= 3,539)** | | |
| --- | --- | --- | --- | --- | --- | --- |
|  | **Odds Ratio** | **95% Confidence Limits** | | **Odds Ratio** | **95% Confidence Limits** | |
| Age* | 0.97 | 0.96 | 0.97 | 0.94 | 0.94 | 0.95 |
| Female | 1.57 | 1.46 | 1.70 | 1.89 | 1.73 | 2.07 |
| **Region (ref: West)** | | | | | | |
| Midwest | 0.87 | 0.77 | 0.97 | 0.79 | 0.70 | 0.90 |
| Northeast | 0.82 | 0.72 | 0.93 | 0.74 | 0.64 | 0.85 |
| Other / Unknown | 1.69 | 0.41 | 7.00 | 1.36 | 0.29 | 6.36 |
| South | 0.94 | 0.85 | 1.04 | 0.88 | 0.79 | 0.98 |
| **Healthcare utilization (in prior year)** | | | | | | |
| No. of physicians* | 1.01 | 1.00 | 1.01 | 1.02 | 1.01 | 1.02 |
| No. of ER visits* | 1.02 | 1.00 | 1.04 | 1.03 | 1.00 | 1.06 |
| No. of unique medications* | 1.01 | 1.00 | 1.01 | 1.02 | 1.01 | 1.03 |
| No. of geriatrician visits* | 0.97 | 0.89 | 1.06 | 0.95 | 0.86 | 1.05 |
| No. of hospitalizations* | 1.00 | 1.00 | 1.01 | 1.00 | 1.00 | 1.01 |
| No. of physician office visits* | 1.01 | 1.01 | 1.02 | 1.01 | 1.01 | 1.02 |
| No. of Rx fills* | 1.00 | 1.00 | 1.00 | 0.99 | 0.99 | 1.00 |
| **Comorbidities (in prior year)** | | | | | | |
| Atrial Fibrillation | 0.57 | 0.53 | 0.62 | 0.36 | 0.33 | 0.40 |
| Alcohol or drug dependence | 1.05 | 0.94 | 1.16 | 1.10 | 0.98 | 1.23 |
| Alzheimer's disease/dementia | 0.91 | 0.80 | 1.03 | 1.04 | 0.89 | 1.22 |
| Ischemic heart diseases | 0.72 | 0.67 | 0.78 | 0.60 | 0.55 | 0.66 |
| COPD | 1.06 | 0.98 | 1.15 | 1.03 | 0.94 | 1.13 |
| Dementia | 0.85 | 0.78 | 0.92 | 0.83 | 0.75 | 0.91 |
| Depression | 0.93 | 0.87 | 1.00 | 1.05 | 0.96 | 1.14 |
| Diabetes | 0.97 | 0.90 | 1.05 | 0.93 | 0.85 | 1.01 |
| Heart failure | 0.60 | 0.55 | 0.65 | 0.43 | 0.39 | 0.47 |
| Hyperlipidemia | 1.03 | 0.94 | 1.12 | 1.04 | 0.94 | 1.14 |
| Hypertension | 0.97 | 0.85 | 1.10 | 0.81 | 0.70 | 0.93 |
| MI | 0.85 | 0.72 | 1.00 | 0.84 | 0.68 | 1.04 |
| Obesity | 1.15 | 1.05 | 1.25 | 1.09 | 0.99 | 1.20 |
| Osteoporosis | 1.02 | 0.92 | 1.13 | 1.07 | 0.96 | 1.21 |
| PVD | 0.91 | 0.83 | 0.98 | 0.91 | 0.83 | 1.01 |
| Rheumatic heart disease | 0.93 | 0.79 | 1.09 | 0.90 | 0.73 | 1.09 |
| Renal dysfunction | 0.80 | 0.65 | 1.00 | 0.67 | 0.51 | 0.87 |
| Sleep Apnea | 0.82 | 0.75 | 0.90 | 0.75 | 0.67 | 0.83 |
| Smoking history | 1.04 | 0.95 | 1.13 | 1.06 | 0.96 | 1.17 |
| Stable Angina | 0.82 | 0.74 | 0.92 | 0.70 | 0.61 | 0.81 |
| Stroke or TIA | 1.13 | 1.00 | 1.28 | 1.12 | 0.97 | 1.29 |
| Unstable Angina | 0.93 | 0.77 | 1.12 | 0.82 | 0.65 | 1.04 |
| Cardiac valve disorder | 0.83 | 0.72 | 0.97 | 0.68 | 0.56 | 0.83 |

* indicates per 1-unit change.

Abbreviations: ER, Emergency Room; Rx, Medical prescription; COPD, Chronic obstructive pulmonary disease; MI, Myocardial infarction; PVD, Peripheral vascular disease; TIA, Transient ischemic attack

**Appendix Table E: Patient characteristics associated with different categories of high anticholinergic burden adjusted for Gagne comorbidity index rather than individual comorbidities.**

| **Characteristic** | **Light vs. Strong** | | | **Mixed vs. Strong** | | |
| --- | --- | --- | --- | --- | --- | --- |
|  | **Odds Ratio** | **95% Confidence Limits** | | **Odds Ratio** | **95% Confidence Limits** | |
| Age* | 1.09 | 1.08 | 1.09 | 1.03 | 1.03 | 1.04 |
| Female | 0.45 | 0.40 | 0.50 | 0.81 | 0.75 | 0.87 |
| **Region (ref: West)** | | | | | | |
| Midwest | 1.49 | 1.28 | 1.73 | 1.19 | 1.07 | 1.32 |
| Northeast | 1.23 | 1.04 | 1.46 | 0.95 | 0.84 | 1.07 |
| Other / Unknown | 0.52 | 0.10 | 2.66 | 0.84 | 0.35 | 1.99 |
| South | 1.22 | 1.07 | 1.39 | 1.10 | 1.01 | 1.20 |
| **Healthcare utilization (in prior year)** | | | | | | |
| No. of physicians* | 0.98 | 0.98 | 0.99 | 0.99 | 0.98 | 1.00 |
| No. of ER visits* | 0.97 | 0.93 | 1.01 | 0.99 | 0.96 | 1.03 |
| No. of unique medications* | 1.09 | 1.08 | 1.10 | 1.09 | 1.08 | 1.10 |
| No. of geriatrician visits* | 1.10 | 0.93 | 1.30 | 1.06 | 0.92 | 1.23 |
| No. of hospitalizations* | 1.00 | 0.99 | 1.00 | 1.00 | 0.99 | 1.01 |
| No. of physician office visits* | 1.00 | 0.99 | 1.01 | 1.01 | 1.00 | 1.01 |
| No. of Rx fills* | 1.01 | 1.01 | 1.01 | 1.01 | 1.01 | 1.01 |
| Gagne comorbidity index* | 1.22 | 1.19 | 1.24 | 1.09 | 1.07 | 1.11 |

* indicates per 1-unit change.

Abbreviations: ER, Emergency Room; Rx, Medical prescription; COPD, Chronic obstructive pulmonary disease; MI, Myocardial infarction; PVD, Peripheral vascular disease; TIA, Transient ischemic attack

**Appendix Table F: Patient characteristics associated with different categories of high anticholinergic burden in patients with >5 prescription fills.**

| **Characteristic** | **Light vs Strong** | | | **Mixed vs Strong** | | |
| --- | --- | --- | --- | --- | --- | --- |
|  | **Odds Ratio** | **95% Confidence Limits** | | **Odds Ratio** | **95% Confidence Limits** | |
| Age* | 1.06 | 1.05 | 1.07 | 1.02 | 1.01 | 1.03 |
| Female | 0.55 | 0.49 | 0.61 | 0.88 | 0.81 | 0.96 |
| **Region (ref: West)** | | | | | | |
| Midwest | 1.36 | 1.17 | 1.58 | 1.16 | 1.04 | 1.28 |
| Northeast | 1.21 | 1.02 | 1.43 | 0.97 | 0.86 | 1.09 |
| Other / Unknown | 0.53 | 0.10 | 2.82 | 0.83 | 0.32 | 2.11 |
| South | 1.17 | 1.03 | 1.33 | 1.08 | 0.99 | 1.18 |
| **Healthcare utilization (in prior year)** | | | | | | |
| No. of physicians* | 0.98 | 0.97 | 0.98 | 0.98 | 0.98 | 0.99 |
| No. of ER visits* | 0.96 | 0.92 | 1.00 | 0.98 | 0.95 | 1.01 |
| No. of unique medications* | 1.09 | 1.08 | 1.10 | 1.10 | 1.09 | 1.11 |
| No. of geriatrician visits* | 1.10 | 0.93 | 1.31 | 1.07 | 0.92 | 1.24 |
| No. of hospitalizations* | 0.99 | 0.99 | 1.00 | 1.00 | 0.99 | 1.00 |
| No. of physician office visits* | 0.99 | 0.99 | 1.00 | 1.01 | 1.00 | 1.01 |
| No. of Rx fills* | 1.01 | 1.01 | 1.01 | 1.01 | 1.01 | 1.01 |
| **Comorbidities (in prior year)** | | | | | | |
| Atrial Fibrillation | 5.68 | 4.77 | 6.77 | 3.10 | 2.64 | 3.63 |
| Alcohol or drug dependence | 1.12 | 0.96 | 1.30 | 1.17 | 1.05 | 1.31 |
| Alzheimer's disease/dementia | 1.00 | 0.80 | 1.25 | 0.92 | 0.76 | 1.12 |
| Ischemic heart diseases | 2.11 | 1.84 | 2.41 | 1.49 | 1.34 | 1.66 |
| COPD | 0.93 | 0.82 | 1.05 | 0.98 | 0.89 | 1.08 |
| Dementia | 1.43 | 1.25 | 1.63 | 1.20 | 1.08 | 1.33 |
| Depression | 1.23 | 1.11 | 1.36 | 1.16 | 1.08 | 1.25 |
| Diabetes | 0.83 | 0.75 | 0.93 | 0.80 | 0.74 | 0.87 |
| Heart failure | 3.12 | 2.65 | 3.68 | 1.80 | 1.56 | 2.08 |
| Hyperlipidemia | 0.87 | 0.78 | 0.98 | 0.89 | 0.82 | 0.96 |
| Hypertension | 1.75 | 1.50 | 2.05 | 1.61 | 1.48 | 1.75 |
| MI | 1.73 | 1.12 | 2.66 | 1.46 | 0.97 | 2.18 |
| Obesity | 0.95 | 0.84 | 1.07 | 1.08 | 0.99 | 1.17 |
| Osteoporosis | 0.91 | 0.79 | 1.05 | 0.93 | 0.84 | 1.03 |
| PVD | 1.11 | 0.97 | 1.27 | 1.00 | 0.90 | 1.12 |
| Rheumatic heart disease | 0.95 | 0.68 | 1.32 | 0.87 | 0.65 | 1.17 |
| Renal dysfunction | 1.11 | 0.74 | 1.66 | 0.87 | 0.62 | 1.24 |
| Sleep Apnea | 1.32 | 1.15 | 1.52 | 1.07 | 0.96 | 1.19 |
| Smoking history | 0.96 | 0.85 | 1.09 | 1.00 | 0.91 | 1.10 |
| Stable Angina | 1.66 | 1.31 | 2.10 | 1.35 | 1.09 | 1.67 |
| Stroke or TIA | 0.89 | 0.73 | 1.08 | 1.00 | 0.85 | 1.18 |
| Unstable Angina | 1.53 | 0.96 | 2.46 | 1.41 | 0.91 | 2.18 |
| Cardiac valve disorder | 1.92 | 1.35 | 2.72 | 1.57 | 1.14 | 2.16 |

* indicates per 1-unit change.

Abbreviations: ER, Emergency Room; Rx, Medical prescription; COPD, Chronic obstructive pulmonary disease; MI, Myocardial infarction; PVD, Peripheral vascular disease; TIA, Transient ischemic attack
